# Tinman Regulates NetrinB in the Cardioblasts of the *Drosophila* Dorsal Vessel

**DOI:** 10.1371/journal.pone.0148526

**Published:** 2016-02-03

**Authors:** Jamshid Asadzadeh, Niamh Neligan, Sunita G. Kramer, Juan-Pablo Labrador

**Affiliations:** 1 Smurfit Institute of Genetics, Trinity College Dublin, Dublin, Ireland; 2 Institute of Neuroscience, Trinity College Dublin, Dublin, Ireland; 3 Department of Pathology and Laboratory Medicine, Robert Wood Johnson Medical School, Rutgers The State University of New Jersey, Piscataway, New Jersey, United States of America; National Institutes of Health (NIH), UNITED STATES

## Abstract

Morphogenesis of the *Drosophila* dorsal vessel (DV) shares similarities with that of the vertebrate heart. Precursors line up at both sides of the embryo, migrate towards the midline and fuse to form a tubular structure. Guidance receptors and their ligands have been implicated in this process in vertebrates and invertebrates, as have been a series of evolutionarily conserved cardiogenic transcriptional regulators including Tinman, the *Drosophila* homolog of the transcription factor Nkx-2.5. NetrinB (NetB), a repulsive ligand for the Unc-5 receptor is required to preserve the dorsal vessel hollow. It localizes to the luminal space of the dorsal vessel but its source and its regulation is unknown. Here, using genetics together with in situ hybridization with single cell resolution, we show how *tin* is required for NetrinB expression in cardioblasts during DV tubulogenesis and sufficient to promote NetB transcription ectopically. We further identify a dorsal vessel-specific NetB enhancer and show that it is also regulated by *tin* in a similar fashion to NetB.

## Introduction

**Fig 1:**
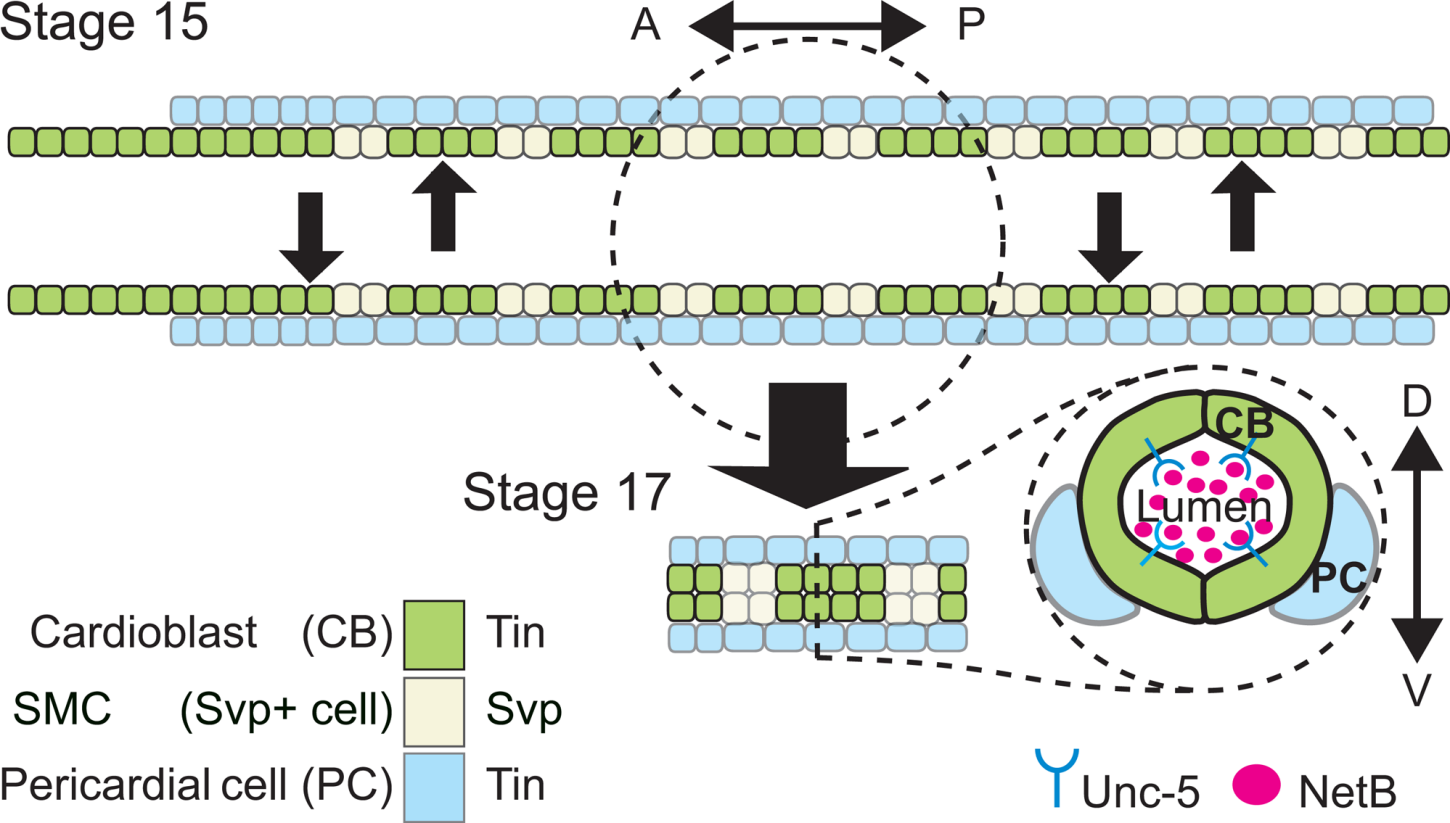
Schematic representation of the *Drosophila* dorsal vessel’s cellular composition and organization. Top, presents *Drosophila* DV at embryonic stage 15 while cardiac progenitors are migrating towards the dorsal midline. Bottom is an schematic representation of the embryonic heart proper at stage 17 when the DV lumen has formed. Aortic portion (right) is oriented anteriorly and the beating (heart) portion (left), posteriorly. Based on the expressed marker TFs, different cell types are colored. CBs are divided into Tin-(green) or Svp-expressing SMCs (light green) subtypes. SMCs of the last three posterior segments of DV make the future ostial cells (inflow valves). CBs are surrounded by pericardial cells (PCs; blue) on their ventrolateral side. PCs are divided into Tin-positive (blue) or Tin-negative PCs (not pictured). Bottom right, is a schematic representation of cross-section view of the DV lumen at stage 17. CBs on the opposite sides assume a crescent-like shape by contacting each other at the two dorsal and ventral apical sides, avoiding contact at the luminal domains, therefore making a hollow space in between. Unc-5 and NetB have been proposed to play a role in the formation of the hollow space between each CB on each row and its counterpart on the opposite row [9].

Heart organogenesis begins with the migration of bilaterally paired groups of cardiac precursors and the formation of a linear tube after they meet [1, 2]. Guidance receptors and their ligands have been implicated at different steps in this process in vertebrates [3–5] and invertebrates [6–10]. In *Drosophila* two rows of bilaterally lined up cardioblasts (CBs) and pericardial cells (PCs) migrate towards the dorsal midline (Fig 1, Stage 15). After migration, the tubular heart (dorsal vessel, DV) forms as CBs fuse, leaving a central luminal space (Fig 1, Stage 17). This complex process is mediated at least through the actions of two guidance systems; the Slit and its Robo receptors [6–8] and NetB behaving as a repulsive cue for the Unc-5 receptor localized at the luminal side of the CBs [9, 10] (Fig 1, stage 17). However, several questions remain unanswered. While NetB is present in the DV lumen it is not clear if it is actually produced by cardiac cells [9, 11, 12]. How these guidance systems are regulated in the DV is also largely unknown. A core network of evolutionary conserved transcription factors (TFs) that regulates heart morphogenesis in both vertebrates and invertebrates, including *Nkx2*.*5* and its *Drosophila* homolog, *tinman* (*tin*), [13] may coordinate their expression playing a later role after cardiac precursor specification. This situation would be analogous to the neuromuscular system where guidance receptors for Slit and Netrin are regulated by TFs that play an earlier role in motoneuron specification [14–16]. In this respect we have recently shown how the Unc-5 receptor is specifically regulated in cardioblasts by *tin* [17].

In this work, we show how *NetB* mRNA specifically localizes in CBs within the DV as does a membrane tethered NetB knocked into the *NetB* locus. NetB is regulated in CBs by *tin* since its mRNA and protein levels are strongly reduced in tin deficient CBs. Furthermore, *tin* is sufficient to induce ectopic NetB expression when misexpressed in the ectoderm. Finally, we identify a NetB DV enhancer that drives expression specifically in CBs within the DV, show that its activity is strictly dependent on Tinman and that Tinman can bind to phylogenetically conserved sites within the enhancer. Together our data shows that *tin* regulates *NetB* in CBs and strongly suggests that *tin* may orchestrate DV assembly, at least partially, through the regulation of a NetB autocrine loop in CBs.

## Results

### NetrinB protein is expressed by Tin-positive cardioblasts in the *Drosophila* dorsal vessel

**Fig 2:**
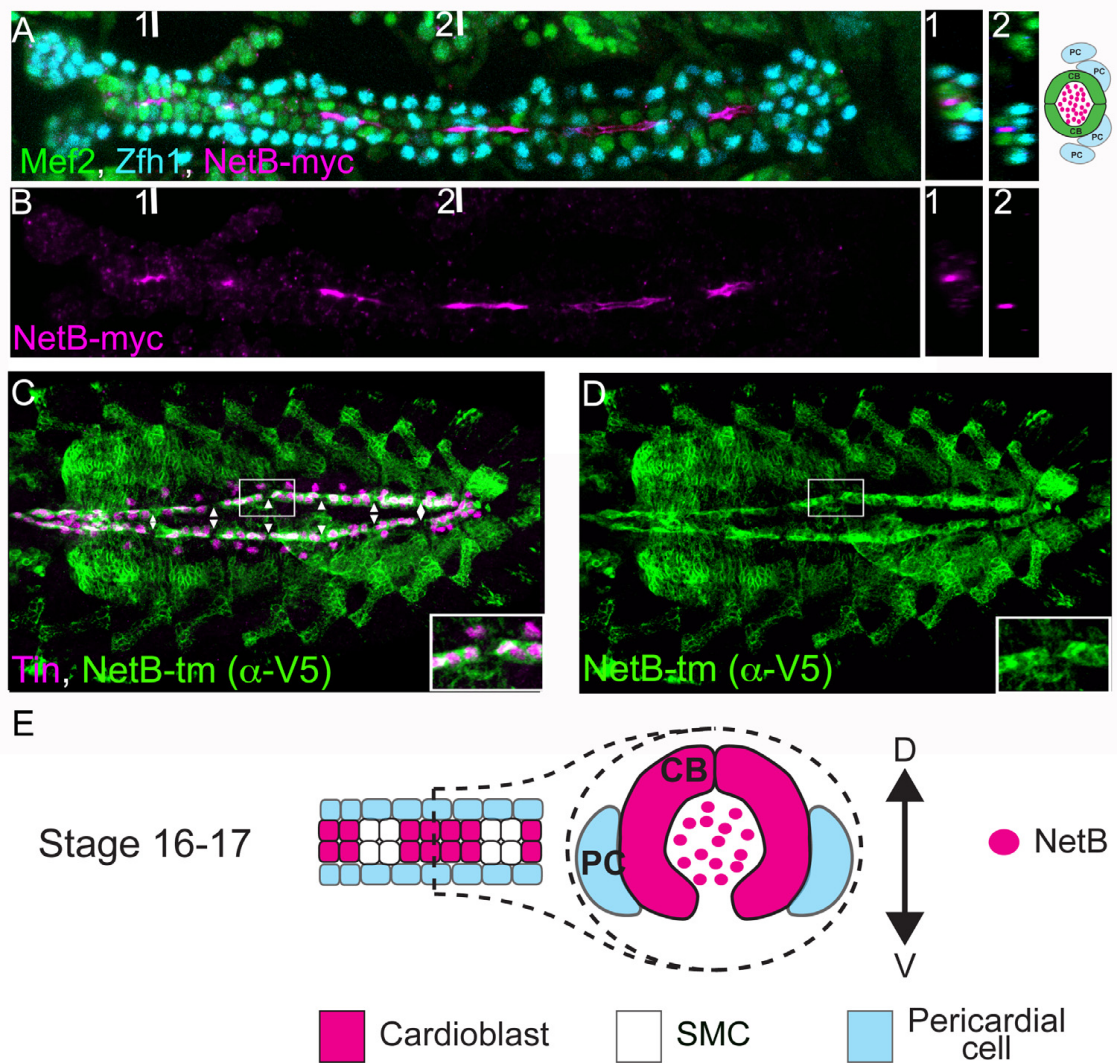
NetB is expressed by CBs and can be detected in the luminal space after dorsal vessel closure. (A and B) Myc staining of embryos expressing a secreted Myc-tagged NetB knock-in [18] (magenta, luminal space) reveals secretion of NetB into the luminal space. CBs and PCs are labeled with a-Mef2 (green) and a-Zfh1 (cyan), respectively. Two cross sections taken from two points numbered 1 and 2 (places of which indicated in A and B) are shown on the right. The cross section at the point 1 is depicted schematically on the right and also in E. Note that due to secretion from CBs, NetB-Myc is mainly concentrated in the luminal space. (C and D) Co-staining for Tin (magenta) and a transmembrane V5-tagged NetB knock-in [18] (green) reveals NetB expression in Tin-positive cardioblasts. Note the regular gaps in NetB-tm expression corresponding to Tin-negative SMCs (arrowheads). All panels are dorsal views with anterior to the left. A magnification of the regions delineated by insets is shown for each panel in A and B.

The Unc-5 receptor and its NetB ligand have been shown to play a role late in tubulogenesis during DV morphogenesis in *Drosophila* [9, 10]. While the receptor is expressed by CBs and present at their luminal side in the DV, NetB accumulates in the lumen from stage 15 onwards [9]. However, *NetB* transcripts were not detected in CBs [9], although other groups have reported its presence in the cardiac mesoderm [11, 12]. In order to determine if cardiac cells produce NetB, we took advantage of NetB knock-in *Drosophila* lines [18]. These lines have been engineered to replace the endogenous *NetB* gene with either a V5-tagged membrane-bound NetB (NetB-tm) or a myc-tagged secreted one. These lines are very useful tools to determine NetB localization and, in particular, the cells where NetB is produced as NetB-tm labels the membranes of NetB expressing cells. In our experiments, we can specifically detect secreted NetB predominantly within the heart lumen in live dissected embryos at late stage 16 (Fig 2A and 2B) as previously reported [9]. When we analyzed NetB-tm expression pattern we could detect it homogeneously expressed in all Tin+ CB membranes but not in Tin–Svp-expressing cells (SMCs) along the DV (Fig 2C and 2D and S1 Fig). Thus, our results show that NetB is produced in CBs, strongly suggesting that CBs are the source of NetB protein in the DV.

### *NetB* mRNA expression in cardioblasts is regulated by *tin*

**Fig 3:**
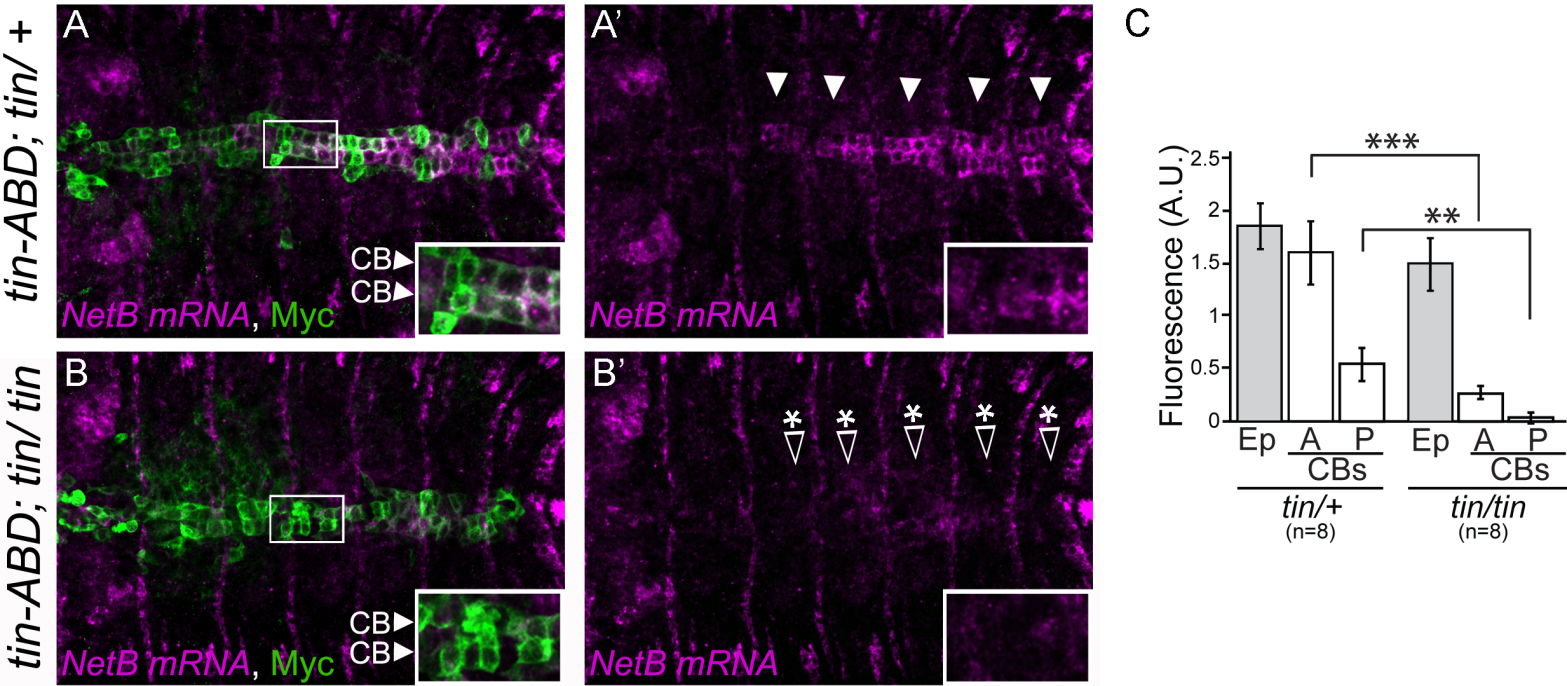
*tin* regulates *NetB* mRNA expression *in vivo*. A and A’ represent *NetB* mRNA (magenta) expression in CBs (green), labeled with Tau-Myc driven by *TinC-Gal4* to label CBs, in heterozygous *tinABD*; *tin346*/*+* embryos (arrowheads). *NetB* expression dramatically drops in homozygous *tinABD*;*tin346*/*tin346* late stage 16 embryos (B and B’; open arrowhead-asterisks in B’ point to where CBs are positioned in B). All panels are dorsal views with anterior to the left. A magnification of the regions delineated by insets is shown for each panel. C, quantification of the in situ signal in cardioblast from the anterior, aorta region (A) or the posterior, heart proper (P) region of the dorsal vessel compared to transversal epidermal signal (Ep) in *tinABD*; *tin346*/*+* and *tinABD*;*tin346*/*tin346* embryos (^***^p<0.003 and ^**^p<0.01).

To confirm that NetB was transcribed in CBs, we performed in situ hybridization in *Drosophila* embryos at late stage 16, which is the onset of DV tube assembly. At this stage, *NetB* mRNA can be readily detected within CBs in the posterior, beating portion of the DV (heart proper) and also, at lower levels within the aorta (Fig 3A and 3A’), which contrasts with the homogeneous expression seen for NetB-tm along the DV (Fig 2). Given that these CBs specifically express the transcription factor Tinman and that we had previously detected NetB in Tin+ CB membranes we wondered if *tin* would be controlling *NetB* expression. *tin* mutants are characterized by their lack of DV, as *tin* is required for early specification of cardiac precursors [19, 20]. Nevertheless, *tin* mutants where *tin’s* expression is restored everywhere except in the DV (*tin-ABD*;*tin*^*346*^/ *tin*^*346*^) are able to form a DV and hatch as adults [21]. To determine whether *tin* regulates *NetB*, we took advantage of these mosaic *tin* mutants to analyze NetB mRNA expression through mRNA in situ hybridization (3B, B’). *NetB* mRNA was significantly reduced when compared with *tin* heterozygous DVs (compare Fig 3A and 3A’ with 3B and 3B’). Thus, not only *NetB* mRNA is transcribed in Tin+ CBs, but also its specific expression pattern matching that of the transcription factor is a consequence of its regulation by *tin* in these cells during DV tubulogenesis.

### NetB protein expression is reduced in *tin* mutants

**Fig 4:**
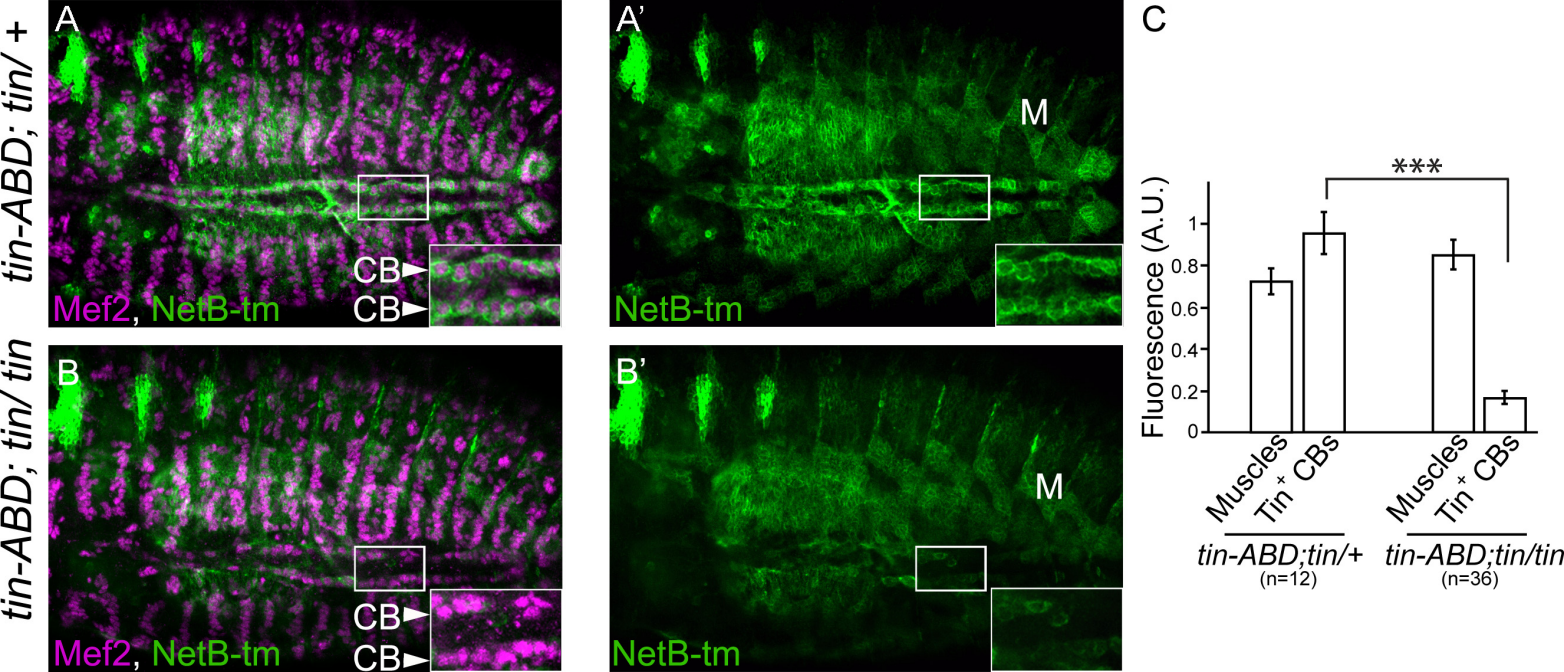
NetB protein expression, in *tin* mutants is downregulated in CBs. As *NetB* mRNA is almost absent in CBs of *tin-ABD*; *tin*^*346*^/*tin*^*346*^ mutants (Fig 3), we tested the NetB-tm protein expression in this background using the transmembrane V5-tagged NetB knock-in [18]. A and A’ represent NetB expression in *tinABD; tin*^*346*^/*+* heterozygous embryos (full confocal stack in S1 Fig). There is no significant difference in the NetB-tm levels between *wild-type* and heterozygotes. However, in *tin-ABD*; *tin*^*346*^/*tin*^*346*^mutants, NetB-tm expression level is dramatically reduced (B, B’, full confocal stack in S2 Fig) while its expression in the muscles [M] is unaffected (C). CBs are labeled with a-Mef2 (Magenta) and NetB is labeled with a-V5 (green). All panels are dorsal views with anterior to the left. A magnification of the regions delineated by insets is shown for each panel (^***^p<2.6x10^-5^).

To determine if NetB mRNA reduction in *tin* mutants would be reflected in NetB protein expression, we analyzed NetB-tm levels in *tin* mutants. As readout of mRNA translation we detected NetB in *tin* heterozygous or mutant CBs and compared its expression levels with NetB expression in muscles. *tin* is not expressed in muscles; therefore muscle levels should remain constant. As expected, NetB-tm is virtually absent in CBs of tin mutants (compare Fig 4A and 4A’ with 4B and 4B’) while muscle expression remains unchanged (Fig 4C).

### Misexpression of *tin* in the ectoderm can induce NetB ectopically

**Fig 5:**
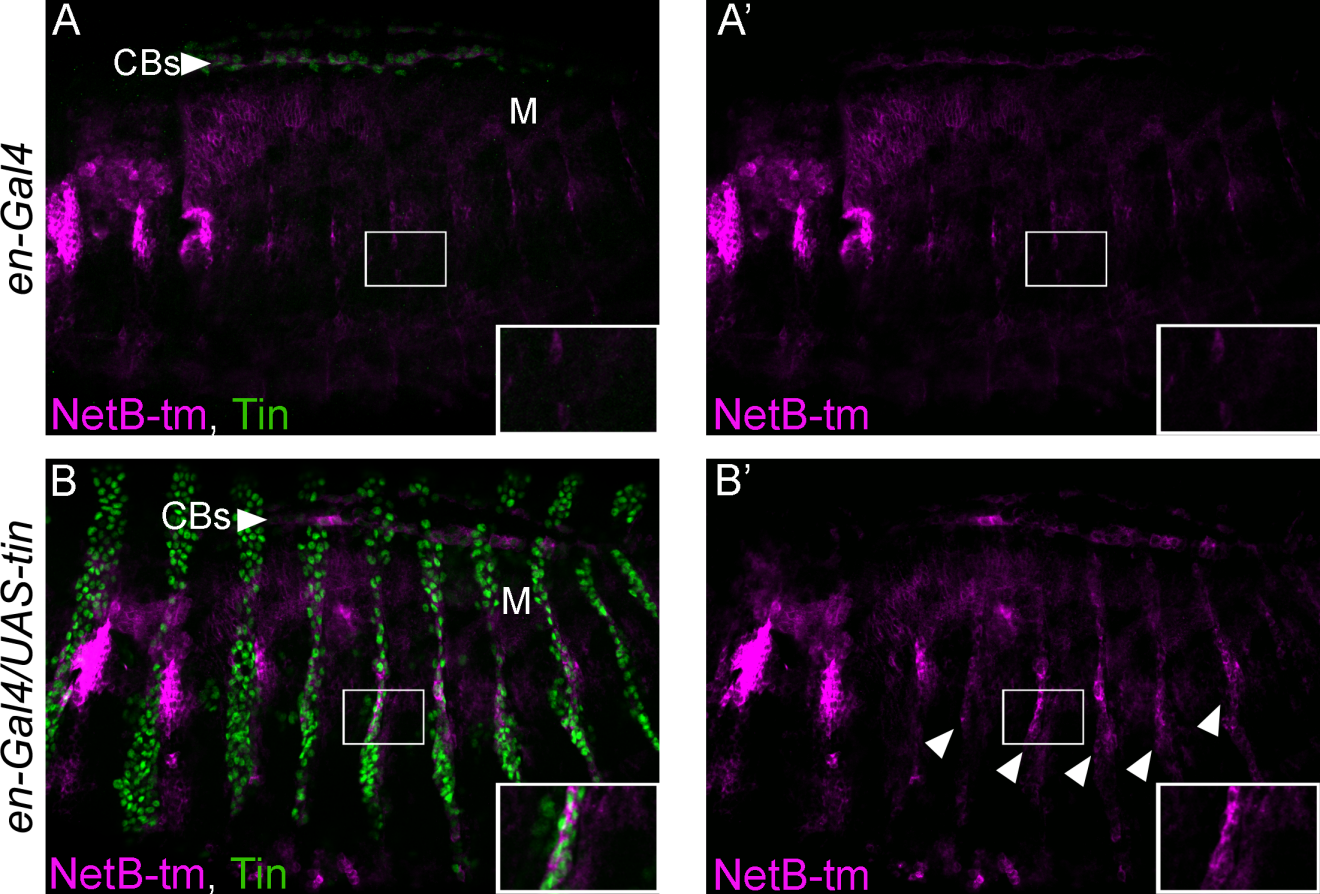
Ectopic expression of Tin induces NetB ectopic expressions *in vivo*. In a wild-type background, staining for NetB protein (magenta), displays little or no NetB expression in the Engrailed stripes (A and A’). However, in an *engrailed-Gal4*>UAS-*tin* background, NetB is strongly induced in Engrailed stripes which now ectopically express Tin (B and B’, arrowheads). For NetB protein staining, the transmembrane V5-tagged NetB-tm knock-in [18] was used. All panels are lateral views with anterior to the left and dorsal side up. A magnification of the regions delineated by insets is shown for each panel.

Given that *tin* is required for NetB expression in the DV, we investigated *tin*’s requirement for its expression *in vivo*. We misexpressed *tin* with an *engrailed-Gal4* driver on a striped pattern in the ectoderm, where neither Tin nor NetB are expressed (Fig 5A and 5A’). Subsequently, we analyzed the induction of NetB and confirmed that ectopic expression of *tin* is sufficient to induce NetB expression (Fig 5B and 5B’) in the characteristic *engrailed* (*en*) striped pattern. These results show that even when Tinman is expressed outside its normal endogenous context it is sufficient to induce expression of NetB. Together, the fact that *NetB* mRNA or protein are almost undetectable in *tin* mutant CBs and *tin’s* ability to induce their expression ectopically in the ectoderm, suggest a direct role of Tinman in the regulation of this ligand.

### A *NetB* enhancer drives expression in cardioblasts

**Fig 6:**
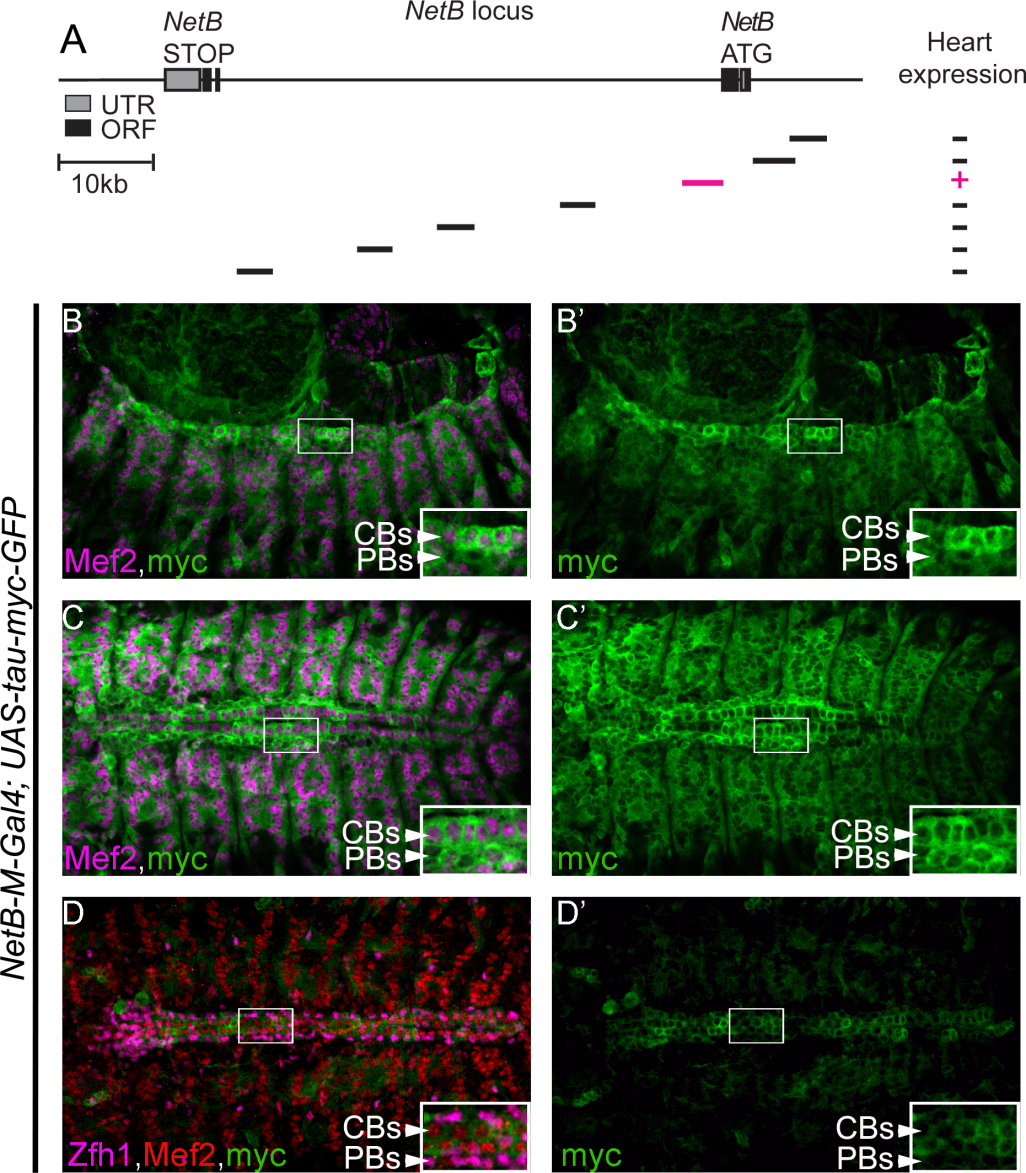
The *NetB*-M reporter is expressed in CBs and weakly in muscles. (A) Schematic representation of the positions and the relative sizes of the available *Gal4* reporters for *NetB* locus. We screened Gal4 from the Janelia collection [22] for DV expression. The line expressing Gal4 efficiently in the DV is shown in magenta. (B-C’) Gal4 expression pattern was examined using a UAS-*Tau-Myc-GFP* construct. Co-staining of embryos with a-Myc (green) and a-Mef2 (magenta) revealed Gal4 expression in muscles and the DV at stage 13 (B, B’) and 15 (C, C’). Co-staining of these embryos with a-Myc (green), a-Mef2 (red) and a-Zfh1 (magenta) reveals a reduction in the expression in other in muscles and pericardial cells while expression in CBs persists at late stage 16–17 (D, D’). All panels are dorsal views with anterior to the left. A magnification of the regions delineated by insets is shown for each panel.

In order to understand how is NetB regulated in the developing DV, we analyzed several enhancers within its locus. Screening the available Gal4 lines from the Janelia collection [22], we identified a fragment (~4kb) within the first NetB intron that can drive expression in the DV at late stages of migration and tubulogenesis (*NetB-M*, Fig 6A). Given the size of the NetB locus and the regions analyzed, this is one potential enhancer element, and we do not exclude the presence of other, potentially more significant, enhancer elements in the locus driving expression in the DV. Nevertheless, the identified enhancer drives expression in mesodermal cells including muscles, pericardial cells and cardioblast at earlier stages (Fig 6B–6C’) and its expression gets restricted at later stages to CBs within the DV (Fig 6B and 6B’). Our UAS-tau-myc-GFP is driven by *NetB-M-Gal4* therefore its expression persists to slightly later stages than the endogenous NetB but faithfully replicates *NetB* transcript and protein expression within the DV (Fig 2C and 2D and Fig 3A and 3A’).

### The *NetB* DV enhancer requires *tin* for its induction in cardioblasts

**Fig 7:**
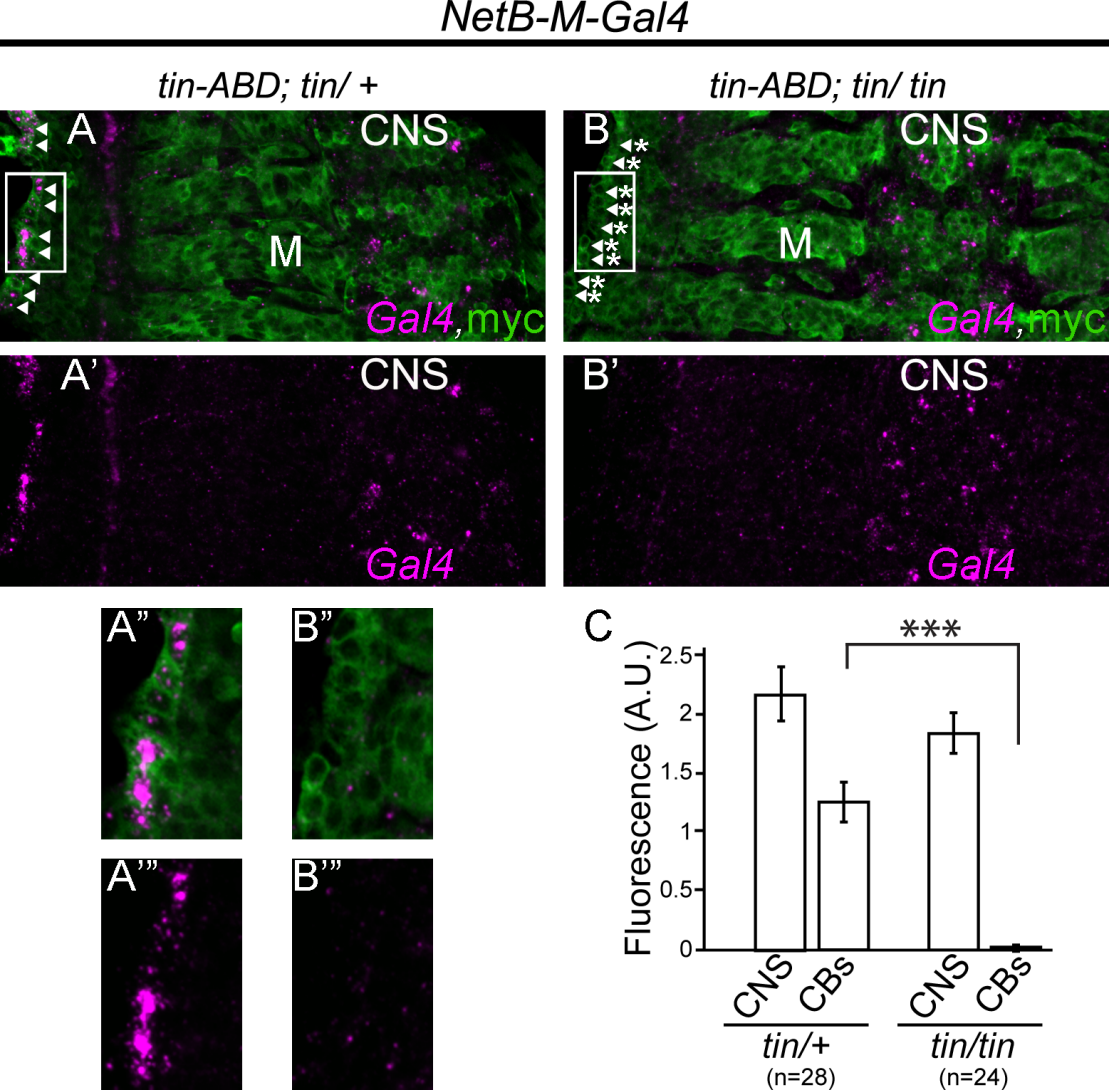
*NetB*-M enhancer element is regulated by Tin *in vivo*. In situ hybridization for *Gal4* mRNA driven by the *NetB-M* enhancer (*Gal4*, magenta) reveals *Gal4* mRNA expression in some cells in the CNS and in the DV in *tin-ABD*; *tin*^*346*^/*+* heterozygous background (A and A’). However, in *tin-ABD*; *tin*^*346*^/*tin*^*346*^ mutant embryos *Gal4* mRNA expression is exclusively absent from the CBs, while CNS cells have the same signal intensity as in heterozygous embryos (B and B’). Reporter driven UAS-tau-myc-GFP is used as a counterstain as it shows a persistent broad expression pattern within the CNS, muscles and DV (myc, green) at stage 15 (A-B”) while the Gal4 mRNA is clearly absent at this stage. Quantification of *Gal4* mRNA expression driven by *NetB*-M element in CBs in *tin-ABD*; *tin*^*346*^/*+* heterozygous versus *tin-ABD*; *tin*^*346*^/*tin*^*346*^ mutant embryos. Fluorescence of *Gal4*-expressing CNS cells is used as internal control (C). While in situ signal in the CNS of heterozygous and mutant embryos remains unchanged (2.15 ± 0.22 s.e.m and 1.83 ± 0.164 s.e.m, in heterozygous or *tin* mutants, respectively), the signal in CBs is significantly dropped from 1.24 ± 0.169 (^***^p<6.8 x 10^−8^) in *tin-ABD*; *tin*^*346*^/*+* heterozygous embryos to 0.0036 ± 0.032 in *tin-ABD*; *tin*^*346*^/*tin*^*346*^ mutants. All panels are views with dorsal side to the left and anterior to the top. A magnification of the regions delineated by insets in A and A’ or B and B’ is shown in A” and A”‘ or B” and B”‘ respectively.

Given that *tin* is required for NetB expression in the DV we argued that the *NetB-M* enhancer may be regulated by *tin in vivo*. To test if *tin* was required to promote transcription through the *NetB* enhancer, we examined if it was able to promote transcription in *tin-ABD*; *tin*^*346*^/*tin*^*346*^ mutant embryos (Fig 7). We analyzed the expression of *Gal4* mRNA under the control of the *NetB-M* enhancer. While *Gal4* was specifically expressed in *tin* heterozygous CBs, we could not detect any mRNA signal in *tin-ABD*; *tin*^*346*^/*tin*^*346*^ CBs (Compare Fig 7A and 7A” with 7B–7B” and 7C). Importantly, reporter expression was not affected in *tin* mutants within the CNS where *tin* is not expressed and therefore, not required.

### Tinman can bind to the *NetB-M* enhancer

**Fig 8:**
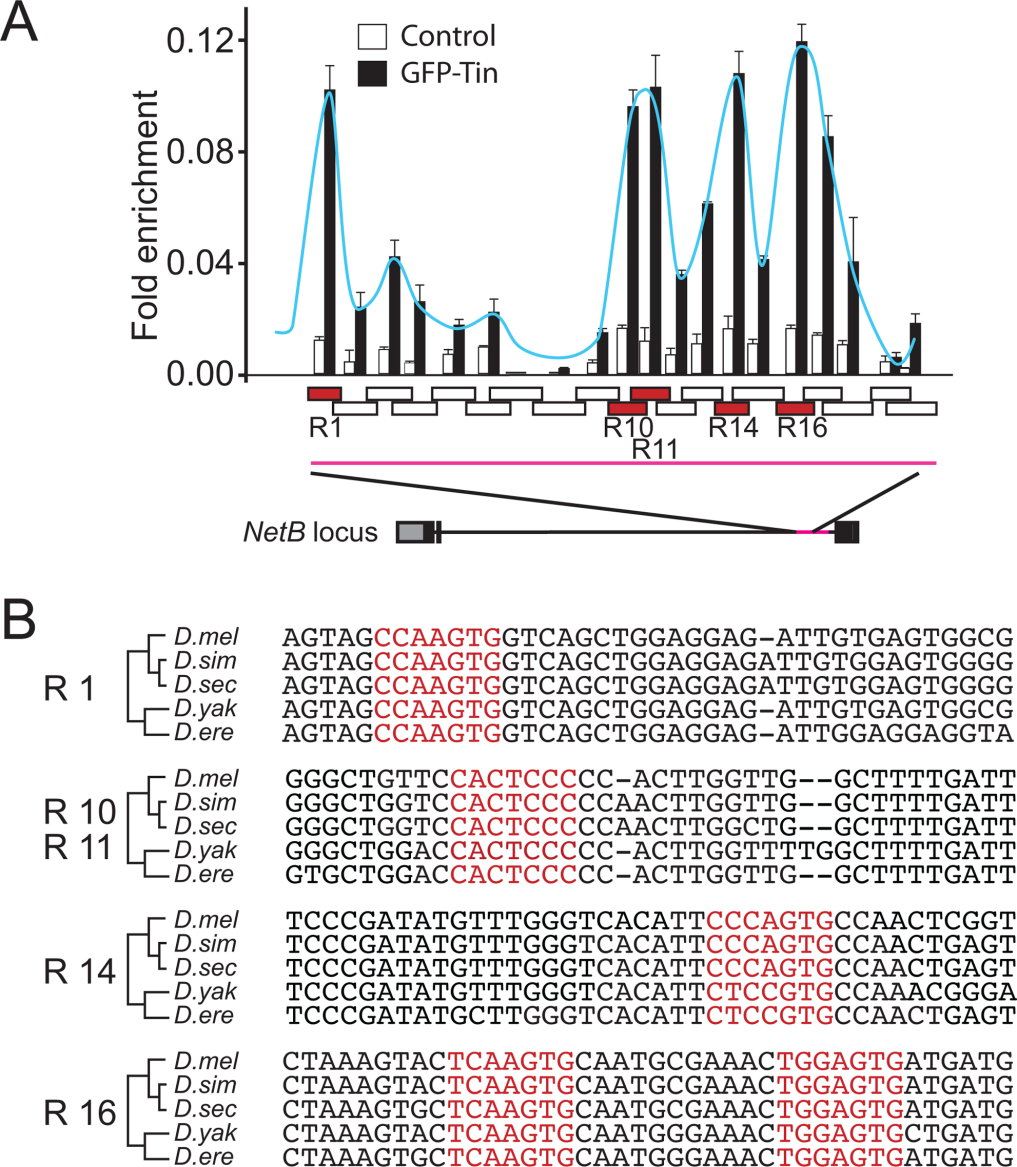
Tin binds directly to NetB-M enhancer element. (A) ChIP analysis of the *NetB-M* genomic loci in S2R+ cells transfected with p*Act5C-GFP-tinman*. The precipitated DNA fragments were amplified by real-time qPCR using overlapping primers (R1-R20) designed over the genomic region covering the *NetB-M* enhancer element. ChIP signal is also illustrated as a curve peaking at R1, R10/R11, R14 and R16. A schematic of *NetB* locus is also illustrated below the graph. Note that the schematic view represents only a ~2kb region (chrX:14,744,635–14,746,673) of the *NetB-M* enhancer that showed significant enrichment as no significant enrichment for the rest of the enhancer was detected above the background. Enrichment is presented as a percentage of total input. (B) Consensus Tin-binding motifs detected with JASPAR within the ChIP enriched region and their conservation in different *Drosophila* species (*mel*, *melanogaster; sim*, *simulans; sec*, *sechellia; yak*, *yakuba; ere*, *erecta*).

Tinman regulates NetB mRNA and protein expression specifically in CBs during tubulogenesis in the DV and it is able to induce its expression ectopically. In addition we have identified a NetB enhancer that drives expression in the DV that is under Tin control in CBs during tubulogenesis. In order to determine if Tin can bind to the NetB enhancer we performed chromatin immunoprecipitation (ChIP) with followed by qPCR using overlapping primers covering the NetB-M enhancer (Fig 8A). We identified 4 peaks of enrichment in the immunoprecipitate indicating that Tin can bind to the NetB enhancer. Further analysis of the sequence revealed phylogenetically conserved Tin consensus binding sequences within each peak that have been preserved across > 10^7^ years of evolution, strongly suggesting its functional relevance (Fig 8B).

Taken together, our data demonstrates that Tinman regulates transcriptionally the *NetB* ligand and a DV enhancer present within its regulatory region specifically in CBs during tubulogenesis. Furthermore, our results also suggest that this regulation may be mediated through a direct interaction of Tin with NetB regulatory regions.

## Discussion

Early dorsal vessel specification events in *Drosophila* take place within the ventral mesoderm. A network of evolutionary conserved cardiogenic transcription factors, including Tinman, integrates their activity to promote DV morphogenesis in the cardiac mesoderm [2, 12]. While Tinman is essential for cardiac cell specification, a later role in the embryonic DV morphogenesis remains largely unexplored. Nevertheless, its function at later stages has been suggested by the defective arrangement of CBs within the DV in cardioblast-specific *tin* mutants [21]. Different guidance systems such as the Slit and its receptors [6–8] and NetB and its Unc-5 receptor [9, 10] are required in late DV morphogenesis for tube assembly. We provide several lines of evidence to demonstrate that *tin* is a regulator of NetB in CBs during DV tubulogenesis: 1- NetrinB is expressed in the DV by CBs and accumulates in a polarized fashion on their luminal side at late embryonic stages (Fig 2); 2- *tin* is required for *NetB* mRNA expression in CBs (Figs 3 and 4); 3- *tin* can induce NetB expression ectopically (Fig 5); and 4- *tin* regulates a *NetB* DV-specific enhancer in CBs (Figs 6 and 7).

NetrinB expression in the DV was not definitely described previously. Its mRNA had been detected in the DV by some groups [11, 12] while another failed to detect it [9] leading to the proposition that NetB was produced in non-cardiac cells and actively or passively transported to the DV lumen. We unambiguously show that NetB is specifically expressed by Tin+ CBs in the DV, strongly suggesting that the NetB source in the luminal side is actually the CBs and it may, therefore, work in an autocrine fashion.

Tissue-specific genome-wide ChIP studies of evolutionarily conserved cardiogenic transcriptional regulators, including Tin, have previously suggested that they may regulate *NetB* through their binding events close to this gene early in cardiac development [12]. Our work analyzes *NetB* regulation by *tin* at later stages when CBs and PCs have been fully specified and shows that *tin* regulates *NetB* and a *NetB* DV enhancer specifically in CBs where *tin* is also expressed. Additionally, we show that Tin can bind to the DV enhancer in phylogenetically conserved Tin binding sites. Full confirmation of their functional relevance would require their mutagenesis and analysis *in vivo*. Thus, our results suggest that Tin specific regulation in CBs may be mediated through a direct binding within the *NetB* locus as a vestige of their embryonic origin in the cardiac mesoderm.

## Materials and Methods

### Genetics

The stocks used in this study were as follows: *Tin-ABD*; *tin*^*346*^*/TM3*, *eve-lacZ*, svp-*lacZ* [21], *en-Gal4*, TinC-Gal4 [23], UAS-*tau-Myc*, Bloomington stocks 49645 (*NetB-M*-Gal4), 49633, 49643, 49645, 49647,49524, 49521, 49346 [22], NetA^∆^ NetBmyc, NetA^∆^ NetBtm [18].

### Immunohistochemistry and mRNA in situ hybridization

Embryo collection, immunohistochemistry and in situ hybridization were performed as previously described [24]. The following antibodies were used for immunohistochemistry: rabbit a-Mef2 (1:2000), guinea pig a-Zfh1 (1:1500) [25], rabbit a-Tin (1:1000) [19], mouse a-V5 (1:1000) (Life technologies; R960-25), chicken a-GFP (1:2000) (Abcam, 13970), mouse a-Myc (1:50) (DSHB). Secondary antibodies: Alexa 555, Alexa 488-conjugated (Invitrogen) and Cy5-conjugated (Jackson ImmunoResearch Laboratories). For *NetB* in situ hybridization, digoxigenin-labeled probes were used as previously described [24]. Probe-hybridized embryos were stained with HRP-conjugated anti-digoxigenin antibody (Roche) overnight. The following day, embryos were incubated with Cy3-conjugated tyramide, as HRP substrate, for 20 (for *NetB* mRNA) or 45 (for *Gal4* mRNA) minutes at room temperature. Zeiss Confocal LSM700 Microscope was used for obtaining stacks of images using 20X or 40X objectives. For fluorescent quantifications, samples were prepared using the same conditions and at the same time, when possible. Image J software was used for image analysis. Background correction was performed for all quantifications. The obtained values from the selected regions were then divided by the values in control areas for each embryo. The normalized values were finally averaged for each genotypic group.

The expression pattern of secreted NetB-Myc was revealed in live-dissected embryos following previously described procedures [18, 26] with minor modifications. Live embryos were staged and propped with their ventral side up, on poly L-lysine-coated glass slides (Fisher Scientific) and were dissected. Following dissection, embryos were fixed in 4% paraformaldehyde in PBS at RT for 15 min and stained with rabbit anti-Mef2, guinea pig anti-Zfh1, and mouse anti-Myc antibodies.

### Chromatin immunoprecipitation

ChIP was performed and analyzed as described previously [17, 27]. Briefly, S2R+ cells transfected with either pAct5C-GFP-Tin or pAct5C (as mock control) were fixed at RT in 1% formaldehyde for 10 minutes. Following cell lysis, the fixed nuclei were sonicated to obtain proper size sheared chromatin. Sonicated lysates were then incubated with rabbit anti-GFP (ab290; Abcam) at 4°C for 2 hours. Lysates were then incubated with protein A-sepharose (P9424; Sigma) for an additional 2 hours. Subsequently, sepharose beads were washed to remove unspecific binding. This step was followed by elution of antibody-GFP-Tin-chromatin from beads at 70°C overnight. The next day, DNA fragments were purified using phenol-chloroform method. The immunoprecipitated fragments were subsequently amplified by real-time qPCR and quantified by comparison to input.

### Statistical analysis

Data are presented as mean values ± s.e.m.. Two-tailed independent samples t-test was used for determining statistical significance for all comparisons between wild-type/heterozygous samples and mutant ones. Histograms were generated using Microsoft Excel 2013.

## Supporting Information

S1 Fig Full stack view of the DV in a *tin-ABD;tin*^*346*^*/+* heterozygous *NetB-tm* knock-in embryo. NetB-tm is expressed in a broad pattern in muscles, gut and dorsal vessel. Individual slice view within a confocal stack reveals a homogeneous expression of NetB-tm along the DV (green). Note the specific expression of NetB-tm in CBs. Panels 1 and 1’ represent the most ventral slice and panels 12 and 12’ represent the most dorsal one. Top panels are double staining with a-Mef2 (magenta) and a-V5 (green). Bottom panels only show the NetB-tm pattern from the corresponding top panel. All panels are dorsal views with anterior to left.
(TIF)

S2 Fig Full stack view of the DV in a *tin-ABD;tin*^*346*^*/tin*^*346*^ mutant *NetB-tm* knock-in embryo. NetB-tm signal is almost absent from individual confocal slices of a *tin* mutant. Note the absence of V5 signal (green) in all CBs. Panels 1 and 1’ represent the most ventral slice and panels 12 and 12’ represent the most dorsal one. Top panels are double staining with a-Mef2 (magenta) and a-V5 (green). Bottom panels only show the NetB-tm pattern from the corresponding top panel. All panels are dorsal views with anterior to left.
(TIF)
