# Supplementary figures and images for: Tinman Regulates NetrinB in the Cardioblasts of the Drosophila Dorsal Vessel
Source: PLoS One. 2016 Feb 3;11(2):e0148526. doi: 10.1371/journal.pone.0148526 (PMC4740434; doi:10.1371/journal.pone.0148526)

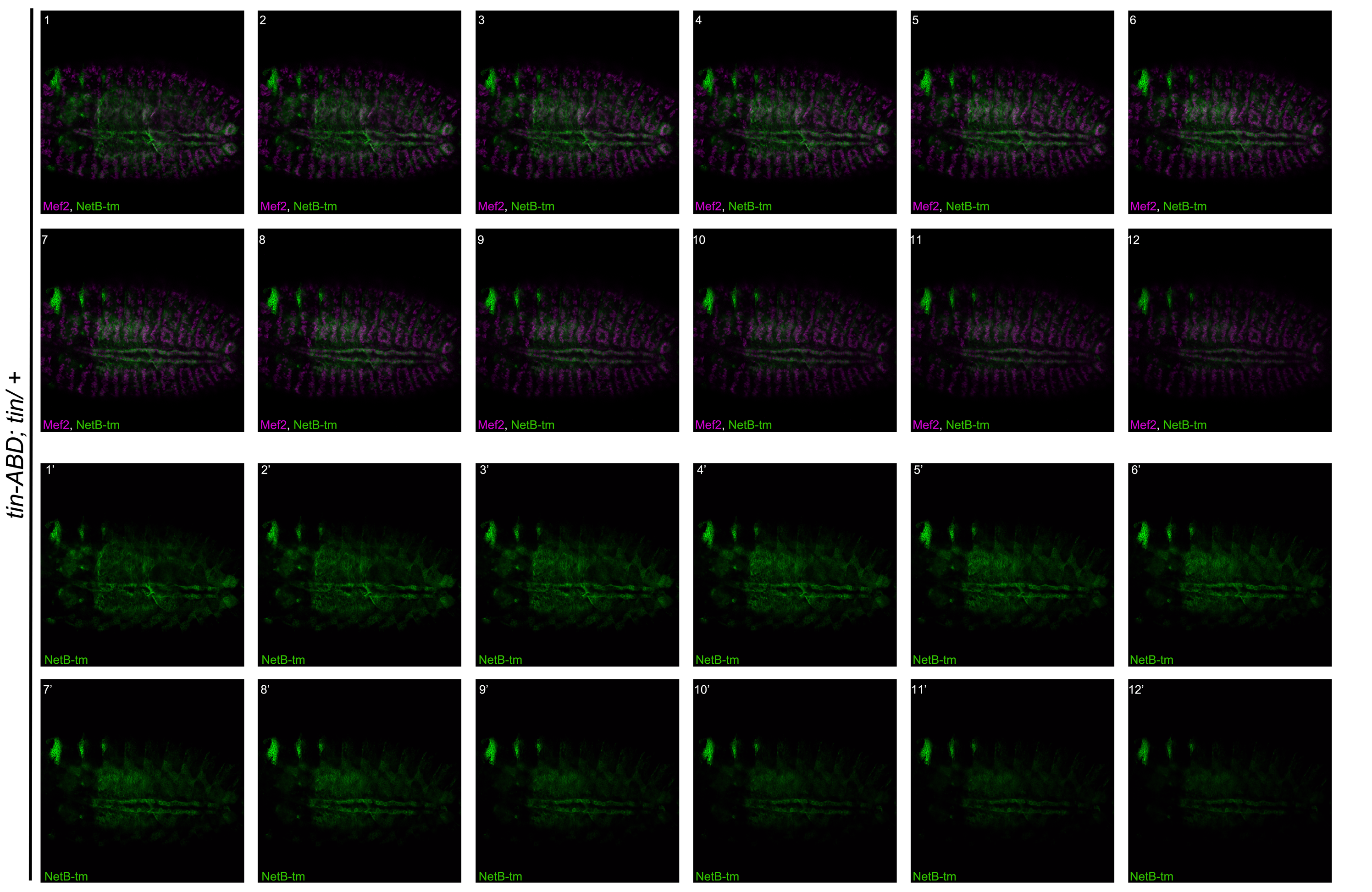

Supplement: S1 Fig — NetB-tm is expressed in a broad pattern in muscles, gut and dorsal vessel. Individual slice view within a confocal stack reveals a homogeneous expression of NetB-tm along the DV (green). Note the specific expression of NetB-tm in CBs. Panels 1 and 1’ represent the most ventral slice and panels 12 and 12’ represent the most dorsal one. Top panels are double staining with a-Mef2 (magenta) and a-V5 (green). Bottom panels only show the NetB-tm pattern from the corresponding top panel. All panels are dorsal views with anterior to left. (TIF) [file pone.0148526.s001.tif]

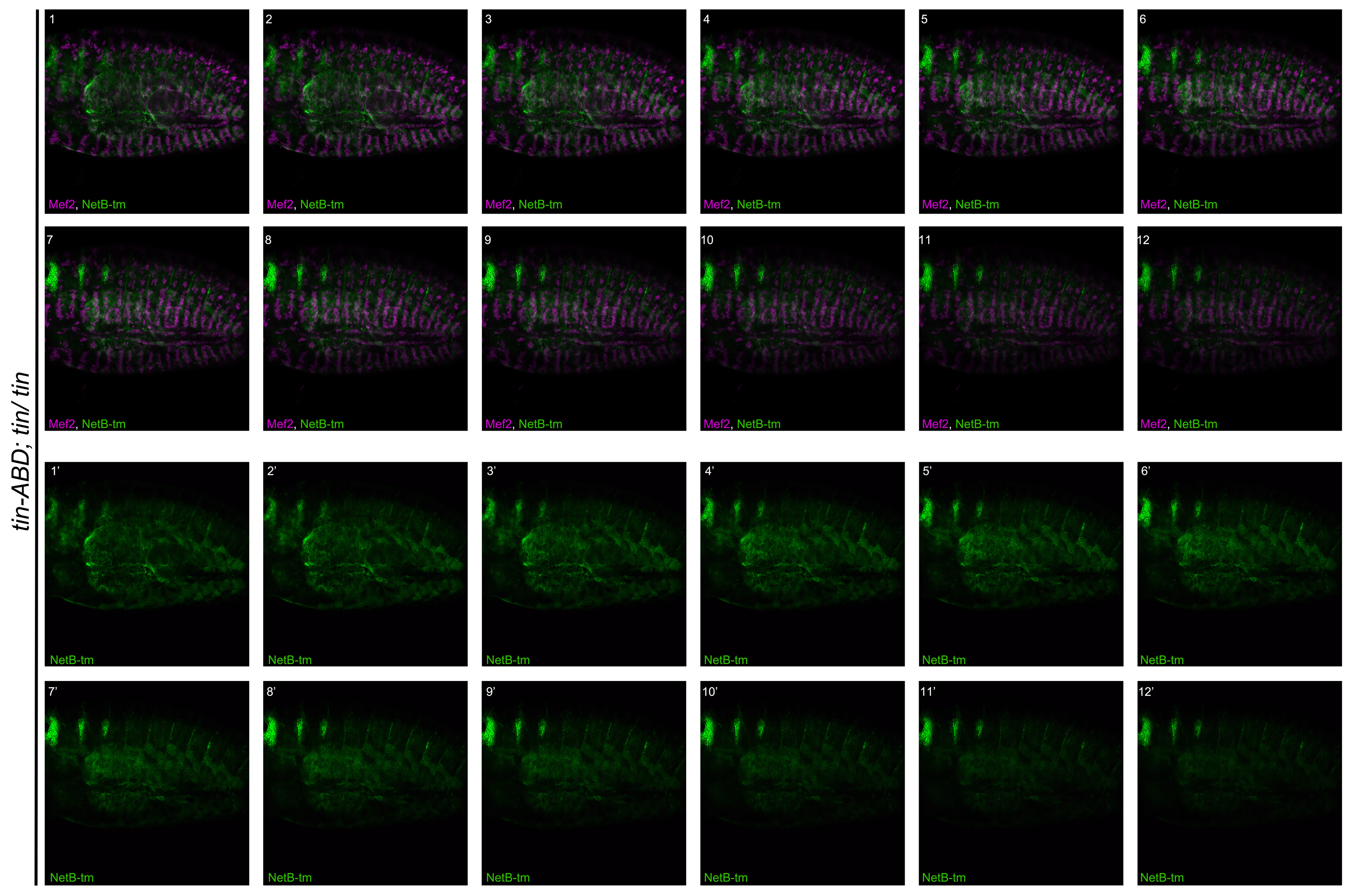

Supplement: S2 Fig — NetB-tm signal is almost absent from individual confocal slices of a tin mutant. Note the absence of V5 signal (green) in all CBs. Panels 1 and 1’ represent the most ventral slice and panels 12 and 12’ represent the most dorsal one. Top panels are double staining with a-Mef2 (magenta) and a-V5 (green). Bottom panels only show the NetB-tm pattern from the corresponding top panel. All panels are dorsal views with anterior to left. (TIF) [file pone.0148526.s002.tif]
